# Bioinspired 3D Bone Model: Mimicking the Cortical–Spongy Bone Architecture and Biology for Enhanced Physiological Representation of Bone

**DOI:** 10.1021/acsbiomaterials.5c00326

**Published:** 2025-07-09

**Authors:** Ana R. Bastos, Lucília P. da Silva, Rui L. Reis, Vitor M. Correlo

**Affiliations:** † 3B’s Research Group, I3Bs—Research Institute on Biomaterials, Biodegradables and Biomimetics, Headquarters of the European Institute of Excellence on Tissue Engineering and Regenerative Medicine, AvePark, Parque de Ciência e Tecnologia, 56059 University of Minho, Zona Industrial da Gandra, Barco, Guimarães 4805-017, Portugal; ‡ ICVS/3B’s—PT Government Associated Laboratory, Braga 4710-057, Portugal

**Keywords:** spongy-like hydrogel, bone models, cortical bone, trabecular bone, vascularization

## Abstract

3D bone models are essential for studying bone physiology, developing therapies, and reducing animal use. Inspired by the hierarchical anatomy of the bone, we shaped previously developed gellan gum–hydroxyapatite spongy-like hydrogels into an outer ring and an inner disc to anatomically represent the cortical and spongy bone, respectively. Biomaterial properties remained stable postmolding, with the polymeric networks absorbing up to 500% of water and exhibiting 4 kPa stiffness. The outer ring was populated with human bone marrow mesenchymal stem cells (HBM-MSCs) and showed the expression of osteogenic genes (ALP/Runx-2/OPN/BSP) by day 14, with ALP and Runx-2 upregulated from day 7 (*p* < 0.5). Models also released angiogenic factors crucial for vasculogenesis, up to an average of 855 pg/mL VEGF, 38 pg/mL bFGF, and 2732 pg/mL Angiopoietin-1. The inner disc, loaded with HBM-MSCs and human dermal microvascular endothelial cells (HDMECs), formed tubular-like structures between days 7 and 14, along with the expression of endothelial cell-specific proteins (CD31/VE-cadherin/vWf) and extracellular matrix components (fibronectin/type I collagen). These effects may have been driven by the release of up to an average of 322 pg/mL VEGF, 243 pg/mL bFGF, and 184 pg/mL Angiopoietin-1. Constructs were then combined and cultured for an additional 7 days, showing a higher release of angiogenic factors in comparison to the independently cultured models, vasculature migration to the cortical-like part, and ALP expression in the spongy-like part. Overall, the 3D vascularized cortical-sponge-like model resembles the intricate cortical and spongy microarchitecture, offering a valuable platform for investigating bone (patho)­physiology and potential therapies.

## Graphical abstract

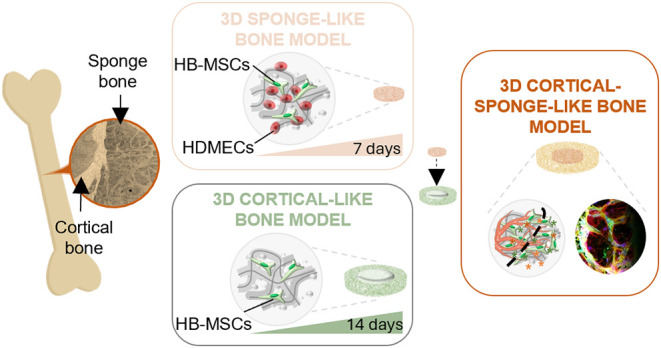

## 1 Introduction

Osteoporosis causes more than 8.9 million fractures annually, resulting in an osteoporotic fracture every 3 s.^1^ This incidence has challenged healthcare systems worldwide, highlighting the critical need for ongoing research and effective treatments. Bone disorders are currently treated with antiresorptive drugs such as bisphosphonates, autologous or allograft bone grafts, bone-graft substitutes, and growth factors. Nevertheless, these treatments exhibit high reintervention rates, morbidity, suboptimal efficacy, and short-lived therapeutic effects, leading to high healthcare costs.^2–4^ 3D bone models serve as valuable platforms for *in vitro* exploration of innovative therapeutics, providing insights into their effects and interactions within the bone microenvironment.^5,6^ Developing 3D bone models that accurately mimic physiological responses observed *in vivo* requires a thorough understanding of bone biology and anatomy, which is essential for designing biomimetic scaffolds that replicate the native structure and function of bone tissue.^7^

The bone is a complex tissue to replicate. Its extracellular matrix (ECM) consists of 30% collagen type I (COL I) fibers and 70% hydroxyapatite (HAp) crystals that enhance the strength and rigidity of the bone.^8^ Calcium and phosphate, the main mineralized components, are deposited onto the collagen fibers, forming HAp crystals. Concerning cellular components, bone consists of osteoprogenitors, osteoblasts, bone lining cells, osteocytes, and osteoclasts.^9,10^ Osteoblasts, one of the most important differentiated cells within the bone, originating from bone marrow mesenchymal stem cells (BM-MSCs), significantly contribute to the structural integrity of bone. They are essential for synthesizing ECM components (COL I, proteoglycans, and noncollagenous proteins), participating in matrix mineralization, and maintaining blood-calcium balance. Osteocytes, the most prevalent and long-lived cells, are mature osteoblasts confined within lacunae, while osteoclasts, originating from mononuclear cells, contribute to bone absorption, calcium regulation, and remodeling. Bone is further categorized into cortical and trabecular (sponge or cancellous) bone.^11,12^ While both types contain osteoblasts, osteoclasts, and osteocytes, they differ in their ECM. Cortical bone exhibits a highly organized and densely packed structure, whereas the sponge bone, located beneath cortical bone, features an open-celled porous foam structure with interconnecting trabeculae that can store substantial energy before yielding.^13^ Sponge bone features porosities from 50% to 90% and contains numerous trabeculae, typically with diameters of 50–300 μm. On the other hand, cortical bone, which makes up 80% of the human skeleton’s weight, has a lower porosity (about 10%) and is much stronger than sponge bone. These structural disparities between the two types of bone lead to significant differences in their mechanical properties. Specifically, the stiffness and yield strength values of human sponge and cortical bone range from 0.1–4.5 to 3–20 GPa and from 2–17 to 33–193 MPa, respectively.^14,15^ Additionally, both compartments have vasculature, although their distribution and density of blood vessels differ.^16^ In cortical bone, the vascular system is primarily situated in Haversian and Volkmann’s canals, delivering oxygen and nutrients to bone cells. Trabecular bone, with higher porosity, has a more abundant distribution of blood vessels, facilitating efficient transport.^17,18^ Therefore, the intricate interaction between bone cells and endothelial cells (ECs) holds crucial importance for bone development, maintenance of homeostasis, and regenerative processes.^19,20^

The replication of bone attributes has been explored by tailoring biomaterials properties. Xiaoyu Wang and co-workers developed a porous tantalum scaffold characterized by wider porosity (60–80%), pore diameter of 200–500 μm, and lower elastic modulus (0.5–4.0 GPa), compared to Zimmer Corporation’s product, which closely mimicked the cancellous bone properties.^21^ Another sophisticated approach aimed to replicate the entire bone, comprising both cortical and trabecular components.^22^ Initially, a zirconia/biphasic calcium phosphate scaffold was employed to mimic trabecular bone. Then, to recreate the Haversian canals and osteon structures, steel wires were bundled with poly­(methyl methacrylate)-poly­(caprolactone-hydroxyapatite) electrospun fibers and then removed. Additionally, the dense application of electrospun fibers was utilized to mimic the cortical bone structure. As a result, the artificial bone exhibited an improved mechanical strength and a porosity level of approximately 70%, comparable to that of natural bone. Most of the developed models aiming to emulate bone complexity and functionality relied on the use of advanced 3D printing technology and polymers resembling natural bone characteristics. For instance, one group replicated the physicochemical environment of cortical bones using 3D printing to investigate tumor phenotypes and pathogenesis.^23^ To closely mimic the characteristics of native cortical bones, the mechanical properties of the 3D-printed poly­(l-lactide) scaffolds, such as stiffness, porosity, and pore size, were adjusted. In particular, the stiffness, which ranged from 100 to 200 MPa, was adjusted according to the interconnected porosity, between 10% and 30%, and the size of large pores (>100 μm). Alternatively, other researchers combined two 3D printing techniques, incorporating thermoplastic polymers (acrylonitrile butadiene styrene and poly­(lactic acid)) with polymeric foam (a mixture of isocyanates and aqueous surfactant), to create scaffolds that imitate bone structures, particularly focusing on the cortico-cancellous interface in human vertebrae.^24^ Advancing the field, a recent study developed 3D-printed biocomposite scaffolds using a multimaterial bioink of silk fibroin, polycaprolactone, fenugreek powder, and bovine bone particles.^25^ The scaffolds demonstrated suitable mechanical strength and *in vitro* bioactivity closely resembling native bone characteristics. These findings support their potential for effective bone tissue regeneration and structural restoration. Despite advancements, most models focus on replicating structural properties and assessing scaffold design’s mechanical performance, with limited evaluation of their biological effects *in vitro*.

Most recently, researchers have undertaken more comprehensive studies that involve both the development of scaffolds and the evaluation of their biological properties *in vitro*. This includes the digital laser processing-based 3D printing of Haversian bone-mimicking Akermanite bioceramic scaffolds featuring cortical and cancellous/trabecular bone structures.^26^ Five different types of scaffolds mimicking Haversian bone were made, varying in the quantity and size of Haversian canals, ranging from 0.8 to 1.6 mm, and included Volkmann canals. The cancellous bone was imitated by a bioceramic meshwork in the central cylindrical hole of the scaffolds. Customizing the scaffold design led to reduced compressive strength and higher porosity as the number of Haversian canals increased. On the other hand, flexural strength increased with more and larger Haversian canals but fewer Volkmann canals. Additionally, employing a multicellular delivery system by co-culturing osteogenic and ECs in the scaffolds showed enhanced *in vitro* osteogenic and angiogenic effects compared to a monocellular delivery system. Furthermore, a novel 3D-printed bilayer scaffold was employed to mimic the microstructure of cortico-cancellous bone.^27^ It showcased precisely controlled architectures with distinct pore sizes and strand thicknesses, comprising both compact and porous layers. The *in vitro* citocompatibility studies confirmed its potential for bone tissue engineering, with increased osteoblastic proliferation and gene, marked by elevated levels of COL I, osteonectin (ON), osteocalcin (OC), and osteopontin (OPN), particularly with a significant increase in OC and OPN observed on day 10. Another group introduced an innovative radial-gradient scaffold with different ink materials, including β-tri-calcium phosphate/polycaprolactone ink via a thermal-assisted extrusion-based 3D printing method, poly­(lactide-*co*-glycolide) via a solvent-assisted low-temperature extrusion-based 3D printing strategy, and gelatin methacryloyl/pure alginate/human mesenchymal stem cells (hMSCs) bioink via a mild bioprinting method.^28^ The fabricated radial-gradient structure revealed a gradual decline in hMSCs numbers from the inner to outer zones in the radial direction. Additionally, on day 1, there was an approximate 78% cell survival rate, with evident cell spreading observed across 3D scaffolds by day 5.

The incorporation of vasculature into 3D bone models is fundamental to replicate the intricate structure of cortical-sponge bone.^29^ Using computational tools and statistical experimental methods, one group studied the optimal experimental conditions for achieving vascularization in bone-mimicking tissues.^30^ Their study identified a combination of parameters, including an endothelial cell density of 3 million cells/ml, a cell ratio of ECs, MSCs, and osteo-differentiated MSCs at 10:1:0, a 1:1 mixture of ECs medium with Angiopoietin-1 (Angio-1) and osteogenic medium, a scaffold design with 2 × 2 × 5 mm^3^ masks, and a hydrogel composed of fibrin (60%) and collagen (40%), as the most effective. In a complementary approach, a recent study combined micro-CT-based modeling with experimental testing to investigate the mechanical behavior of peacock feather shafts, illustrating how biologically optimized hierarchical architectures can inspire the development of robust, lightweight, and multifunctional scaffolds.^31^ This underscores the value of integrating bioinspired designs and computational strategies in the future optimization of both the scaffold architecture and vascular network formation. Other groups focused on designing 3D scaffolds to support co-cultivation of human dermal microvascular endothelial cells (HDMECs) with bone cells, with the goal of advancing insights into cellular crosstalk and their role in tissue regeneration and vascularization, crucial for replicating *in vivo* microenvironments.^32,33^ Their results indicated that the co-cultivation of osteoblasts and HDMECs in direct contact within 3D scaffolds led to the formation of structures resembling blood vessels, upregulation of genes associated with osteogenesis, increased vascular endothelial growth factor (VEGF) secretion, and expressed the gap junction protein connexin 43, ensuring heterotypic communication between the two cell types.^32,33^

Despite advances in the development of 3D bone models that better replicate the architecture and biology of both cortical and spongy bone, a comprehensive understanding of the interactions between these two components remains lacking. Specifically, it is still unclear how the cortical compartment influences the spongy region and vice versa. More importantly, the impact of vascularization on osteogenesis in the cortical bone—as well as the reciprocal effect of osteogenesis on vascularization—is not yet well understood. Addressing these questions requires the development of models with clearly defined and separable cortical and spongy regions that can be studied independently or in combination after assembly. Such models would enable targeted investigation of region-specific bone biology and, in the future, could facilitate the development of drugs tailored to each bone compartment. A deeper and more integrated understanding of cortical-spongy bone interactions is essential for advancing regenerative strategies and bone tissue engineering. Thus, our objective was to develop a 3D bone model that replicates the architecture and biology of cortical-sponge bone and to understand the interaction between these two compartments. To this aim, two distinct 3D models representing cortical and sponge-like bone structures were developed separately. Accordingly, spongy-like hydrogels, consisting of gellan gum (GG) and HAp, were prepared and shaped into a ring and a small disc. For the cortical-like bone model, human bone marrow mesenchymal stem cells (HBM-MSCs) were cultured within rings, while for the 3D sponge-like bone model, HDMECs were co-cultured with HBM-MSCs small discs. The specific time point at which bone tissue maturation occurs in the 3D cortical-like bone model was assessed by measuring the expression levels of osteogenic markers, while the time needed for vasculogenesis initiation in the 3D sponge-like bone model was determined by measuring the expression of characteristic endothelial phenotype markers. The release of angiogenic growth factors was also evaluated in both 3D cortical and sponge-like bone models. The final 3D cortical-sponge-like bone model was prepared by coculturing the 3D cortical-like and sponge-like bone models together and characterized to infer its resemblances with the native bone.

## 2 Materials and Methods

### 2.1 Preparation of GG-HAp Spongy-Like Hydrogels

GG-HAp spongy-like hydrogels composed of GG and HAp were prepared following a preestablished procedure.^34–36^ In summary, Gelzan powder (1.25% w/v) (Sigma-Aldrich, Missouri) was dissolved at 90 °C in a water bath with stirring for 30 min. Further, the water bath was cooled to 60 °C, and HAp powder (10% (w/v), Plasma Biotal, Buxton, U.K.), and CaCl_2_ (0.18% (w/v), Sigma-Aldrich, Missouri) was added to the GG solution. The resulting GG-HAp solution was cast into a Petri dish and left at room temperature (RT) for 30 min for cross-linking. Afterward, 8 mm diameter and 3 mm thick discs were punched from the GG-HAp hydrogel and incubated in phosphate-buffered saline (PBS, Sigma-Aldrich, Missouri) for 1 day for further cross-linking and stabilization. Then, GG-HAp hydrogels were frozen overnight at −80 °C, followed by a 3-day freeze-drying process. Dried polymeric networks were sterilized by ethylene oxide (3 h, 50–52 °C) and further punched in the middle with a 4 mm punch to attain a ring (8 mm of outside diameter and 4 mm of inner diameter) and a small disc with 4 mm of diameter.

### 2.2 Fourier Transformed Infrared Spectroscopy

GG, GG-HAp, and Fibronectin (FN)-coated GG-HAp dried polymeric networks were analyzed by Fourier transform infrared (FTIR) using the IRPrestige-21 instrument (Shimadzu Corporation, Kyoto, Japan), employing the attenuated total reflectance (ATR) technique. The analysis was conducted in transmittance mode across the spectral range of 500 to 4000 cm^–1^, with a resolution of 4 cm^–1^ and 32 scans. A baseline correction was performed prior to the analysis of samples.

### 2.3 Scanning Electron Microscopy

Scanning electron microscopy (SEM, JSM-6010 LV, JEOL, Tokyo, Japan) was used to assess the microstructure of the dried polymeric networks of GG-HAp mimicking the cortical-sponge bone architecture. Samples were sputter coated with platinum and observed by SEM at an accelerating voltage of 10 kV.

### 2.4 Water Uptake

The GG-HAp dried polymeric networks, prepared in various geometries (ring, small disc, 3D model, and control disc), were initially weighed in their dry state (Wd) before being immersed in PBS at 37 °C to become spongy-like hydrogels. During this period, at specific time points (30 min, 3, 24, 48, and 72 h), the spongy-like hydrogels were weighed in their wet/swollen state (Ww). The water uptake percentage over time was then calculated using the following equation

$$water\;uptake\left(\%\right)=\frac{\left(Ww-Wd\right)}{Wd}\times 100$$

### 2.5 Compressive Testing

Compressive stiffness was determined using an Instron 5543 universal mechanical testing machine (Instron Int. Ltd). Before testing, GG-HAp dried polymeric networks were incubated at 37 °C in a PBS solution for 24 h to become spongy-like hydrogels. Samples were used both in their initial disc form (control) and after being punched into the two geometries used in this study: rings and small discs. Testing was performed at RT with a preload force of 0.01 N and a strain rate of 0.5 mm/min, reaching a maximum strain of 40%. The compressive modulus was calculated by analyzing the linear region (0%–5% strain) of the stress–strain curve. Results, presented as mean ± standard deviation (SD), were obtained from 6 samples.

### 2.6 *In Vitro* Studies

#### 2.6.1 Cell Types and Culture

Primary HBM-MSCs were purchased from Biopredict (Bizkaia, Spain) and grown in an alpha­(α)-MEM culture medium supplemented with 10% fetal bovine serum (FBS) and 1% antibiotic/antimycotic (all from Alfagene, Portugal). Cells were used until passage 5. The osteogenic α-MEM medium was prepared by adding 10^–7^ M dexamethasone, 0.01 M β-glycerophosphate, and 50 μg/mL ascorbic acid (all from Sigma-Aldrich, Missouri) to the α-MEM medium.

HDMECs were purchased from Lonza and cultured in Microvascular Endothelial Growth Medium-2 (EGM-2 MV) (all purchased from Lonza, Switzerland). Cells were used in passages 3–5. Furthermore, for cell culture experiments, the EGM-2 MV was supplemented with osteogenic growth factors, namely, 10^–7^ M dexamethasone and 0.01 M β-glycerophosphate (all from Sigma-Aldrich, Missouri).

#### 2.6.2 Cell Entrapment within GG-HAp Spongy-like Hydrogels

A 15 μL droplet containing a suspension of 400,000 HBM-MSCs was dispensed in the GG-HAp dried polymeric network ring. The construct was incubated for 30 min in standard culture conditions (37 °C and 5% CO_2_), and then 1 mL of osteogenic α-MEM medium was added to each 3D construct. HBM-MSCs loaded in GG-HAp spongy-like hydrogel rings were left in culture for up to 21 days and analyzed at 7, 14, and 21 days.

A 15 μL droplet containing a suspension of 640,000 HDMECs and 160,000 HBM-MSCs was carefully seeded onto FN-coated small disc spongy-like hydrogels. The coating was performed by adding a 15 μL droplet of FN (Thermo Fisher) at a concentration of 10 μg/mL, followed by incubation at 37 °C for 1 h. This was done to allow a maximum HDMECs entrapment, as previously described.^37^ After the seeding, cells were allowed to adhere/entrap for 30 min at 37 °C in an environment with 5% CO_2_ prior to addition of the osteogenic EGM-2 MV medium. HDMECs/HBM-MSCs loaded in GG-HAp spongy-like hydrogels small discs were left in culture for up to 14 days and analyzed at 7 and 14 days.

The 3D model was prepared by transferring the HDMECs/HBM-MSCs-laden GG-HAp spongy-like hydrogel small discs precultured for 7 days into the HBM-MSCs-laden GG-HAp spongy-like hydrogel rings precultured for 14 days. The system was left in culture in osteogenic EGM-2 MV medium for further 7 days and analyzed.

#### 2.6.3 RNA Isolation and Real-Time Polymerase Chain Reaction (RT-PCR)

The expression of bone-related genes (alkaline phosphatase (ALP), runt-related transcription factor 2 (Runx-2), COL I, and OPN) was quantified by RT-PCR in the GG-HAp spongy-like hydrogel rings after 0 (30 min postseeding/entrapment), 7, 14, and 21 days of cell culture. Briefly, 600 μL of TRIzol reagent (Alfagene, Portugal) was added and stored at −80 °C for later use. Subsequently, total mRNA isolation was carried out using the Direct-zol RNA MiniPrep kit (Zymo Research, California) in accordance with the manufacturer’s guidelines. The RNA concentration and purity were assessed using a NanoDrop ND-1000 spectrophotometer (NanoDrop Technologies, Wilmington, DE). To synthesize cDNA, 100 ng of the total RNA was subjected to reverse transcription, resulting in the creation of single-stranded cDNA. This process was carried out using the qScript cDNA SuperMix synthesis kit (VWR, Leuven, Belgium) and the Eppendorf Mastercycler ep realplex gradient. The expression of bone-related genes listed in Supplementary Table S1 was assessed by an RT-PCR Mastercycler Realplex machine (Realplex, Eppendorf, Germany). To that, a PerfeCTAR©SYBR Green FastMix kit (Quanta BioSciences) was used according to the manufacturer’s instructions with the reaction conditions consisting of an initial incubation at 95 °C for 2 min, followed by 45 cycles of denaturation (95 °C, 10 s), annealing (specific to each gene, 25 s), and extension (72 °C, 30 s). The Livak (2^–ΔΔCt^) method was employed to calculate the expression data for transcripts. This involved normalizing the target value to the glyceraldehyde-3-phosphate dehydrogenase (GAPDH) housekeeping gene, followed by a second normalization by samples collected on day 0. Three independent experiments were performed (*n* = 3), each including three replicates per condition and technical duplicates for each replicate.

#### 2.6.4 Immunocytochemistry (ICC)

Immunocytochemistry was used to observe the expression of bone (ALP, Runx-2, COL I, OPN, and Bone sialoprotein (BSP)) and endothelial (cluster of differentiation 31 (CD31), FN, VE-cadherin, and vWf (Von Willebrand factor))-related proteins, respectively. After each time point (7, 14, and 21 days), GG-HAp spongy-like hydrogels were fixed in 10% (v/v) formalin (Sigma-Aldrich, Missouri) for 1 h at RT. Next, 3D constructs were cut into small sections (∼5 × 2 mm), treated with 1% (v/v) Triton-X 100 (Sigma-Aldrich, Missouri) for 20 min on ice, and then blocked with 2.5% (w/v) horse serum (Vector Laboratories, S-2012) for 1 h. Following this, cell-laden spongy-like hydrogels were incubated with primary antihuman antibodies (Supporting Table S2) diluted in a 0.2% (v/v) Triton-X 100 solution 16–24 h at 4 °C. After thoroughly washing with PBS for 3–4h with PBS replenishing every 30 min, under stirring conditions, the samples were incubated overnight at 4 °C with the secondary antibodies, specifically Goat Anti-Mouse IgG H&L (Alexa Fluor 488) and/or Donkey Anti-Rabbit IgG H&L (Alexa Fluor 594) (Abcam, U.K.), at a dilution of 1:500 in a 0.2% (v/v) Triton-X 100 solution. Additionally, nuclei were counterstained with 0.04 mM Hoechst (Alfagene). Finally, samples were assessed using a confocal laser scanning microscope (TCS SP8, Leica, Mannheim, Germany) at a 20× magnification. The number of vessels and nodes, as well as the average vessel diameter, were quantified from 10 CD31-immunostained images using ImageJ. Due to the heterogeneity of vessel distribution across biomaterial, results are reported as the total number of vessels or nodes normalized to the total imaged area.

#### 2.6.5 Protein Extraction and Western Blot (WB)

Western Blot analysis was employed to conduct a semiquantitative and qualitative assessment of bone-related proteins (ALP and Runx-2). The expression of bone-related proteins was normalized using the GAPDH housekeeping gene. 3D cell-laden spongy-like hydrogels were collected at each time point (0, 7, 14, and 21 days) and placed at −80 °C for subsequent analysis. To initiate the protein extraction process, 100 μL of RIPA buffer (Sigma-Aldrich, Portugal) was added to each sample, and the mixture was macerated and incubated on ice for 30 min. Further, samples were centrifuged at 10,000*g* for 20 min at 4 °C, and the supernatant containing the protein extract was transferred to new vials. Then, the Micro BCA Protein Assay Kit (Alfagene, Portugal) was performed according to the manufacturer’s instructions to determine the protein concentration of each sample. After that, the SDS-PAGE was assessed by loading 20 μL equivalent of each sample onto a Bolt 8% Bis-Tris Plus gel (Alfagene, Portugal), which was subsequently transferred to a PVDF membrane (Alfagene, Portugal). Further, nonspecific staining was blocked with a 5% (w/v) bovine serum albumin (BSA) solution in tris-buffered saline with 0.1% (v/v) Tween 20 (TBS-T) (all from Sigma-Aldrich, Portugal) for 1 h in order to identify only the expression of bone-related proteins. The blots were then exposed to different antibodies (Supporting Table S3) overnight at 4 °C. Following this, the blots were washed with TBS-T and incubated at RT for 1 h with antirabbit/mouse IRDye 800CW/680RD secondary antibodies (1:10 000, Sigma-Aldrich, Portugal) to detect the bounded antibodies. The bone-related proteins were visualized using the Odyssey Fc Imaging System (LI-COR) in the 700 or 800 channel, and the bands were imaged and quantified using Image Studio Software (LI-COR). All blots and gels (*n* = 3) were processed simultaneously in a total of 3 experiments.

#### 2.6.6 Luminex

The analysis of Von Willebrand Factor A2 (vWF-A2), platelet-derived growth factor BB (PDGF-BB), VEGF, basic fibroblast growth factor (bFGF), Angio-1, OPN, bone morphogenetic protein 2 (BMP-2), and bone morphogenetic protein 4 (BMP-4) proteins in cell culture supernatants was carried out using a Human Magnetic Luminex Screening Assay (R&D Systems), following the instructions provided by the manufacturer. First, samples that had been stored were thawed, centrifuged at 16,000*g* for 4 min, transferred to new vials, and then prepared for use by making a 2-fold dilution with Calibrator Diluent RD6–52. The standard cocktails included in the kit were reconstituted and mixed to create a concentrated Standard 1 using the Calibrator Diluent RD6–52. A series of 3-fold dilutions were created from Standard 1 by using the Calibrator Diluent RD6–52, which was used as a blank. After preparing the samples and standards, 50 μL of each sample/standard was added to each well, followed by 50 μL of a microparticle cocktail, prepared using the Calibrator Diluent RD2–1. The plate was then incubated for 2 h at RT on a horizontal orbital microplate shaker (VWR) set at 800 rpm. Following this incubation, three washing steps were carried out using the wash buffer, and a magnetic device designed for the microplate was employed for this purpose. Next, 50 μL of biotin-antibody cocktail prepared in Calibrator Diluent RD2–1 was added to each well, and the plate was incubated for an additional 1 h at RT on the same microplate shaker set at 800 rpm. After this incubation, the samples were washed again, as previously described. Subsequently, 50 μL of streptavidin-PE, prepared in Wash Buffer, was added to each well and incubated for 30 min at RT on the horizontal orbital microplate shaker. Following this incubation, the samples were washed again, as previously described. Then, 100 μL of wash buffer was added to each well, and the microplate was shaken for 2 min on the horizontal orbital microplate shaker. Finally, the plate was read using the Luminex MAGPIX Instrument System (MAGPIX, Thermo Fisher Scientific, U.K.). Each experiment was performed with biological triplicate (*n* = 3), and each measurement was taken in duplicate. The concentration of each analyte was calculated using Luminex xPONENT 4.2 software.

#### 2.6.7 Statistical Analysis

GraphPad Prism 8.0 software was used for conducting statistical analysis studies. The normal distribution of the data was assessed using the Shapiro–Wilk normality test. Statistical significance was determined based on the distribution of the data. For normally distributed data, one-way ANOVA followed by Tukey’s multiple comparisons post-test, or the unpaired two-tailed Student’s t test, were employed. Data that did not follow a normal distribution were analyzed using the Mann–Whitney U test or the Kruskal–Wallis test followed by Dunn’s multiple comparison post-test. Statistical significance was established at the following levels: *p < 0.05, **p < 0.01, ***p < 0.001, and ****p < 0.0001. All data are presented as the mean ± SD and were performed in triplicate or more, as applicable. Data analyses were performed in a blinded and randomized manner to minimize bias.

## 3 Results

Spongy-like hydrogels encompassing GG, HAp, and CaCl_2_ as cross-linking agents were developed as previously reported^37^ but with an additional coating of FN for HDMECs adhesion (Figure 1).

**Figure 1:**
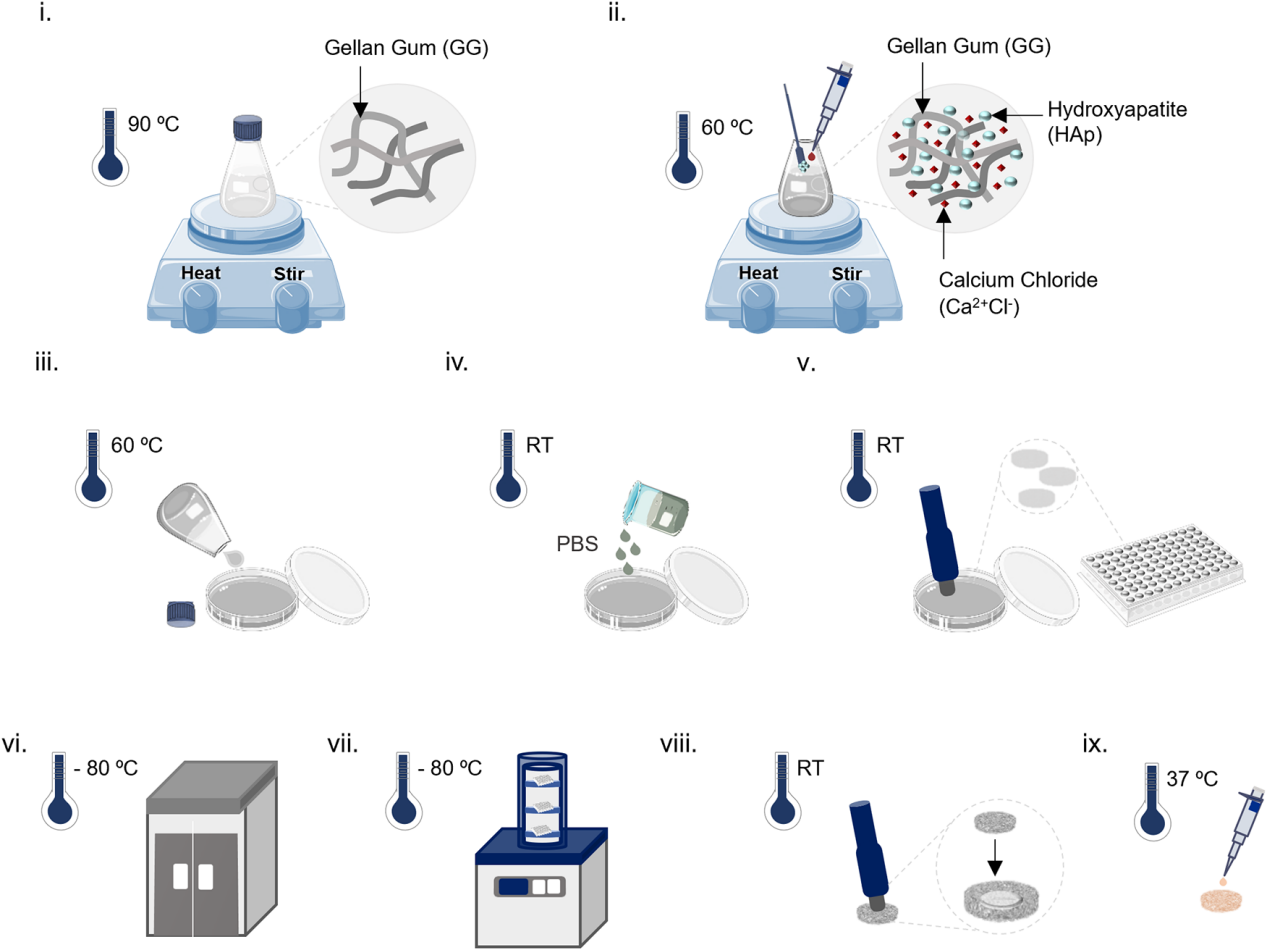
Schematic representation of GG-HAp spongy-like hydrogels fabrication into cortical-like and sponge-like bone parts: (i) dissolution of GG at 90 °C with stirring; (ii) decrease of temperature to 60 °C, and addition of HAp and CaCl_2_, under stirring; (iii) casting the GG-HAp solution into a Petri dish; (iv) formation of the GG-HAp hydrogel with the decrease of temperature, and addition of PBS for further ionic cross-linking; (v) punching the GG-HAp hydrogel into smaller discs (8 mm of diameter, 3 mm of thickness) and transference to a multiwell plate; (vi) freezing the samples within the multiwell plates with samples at −80 °C, overnight; (vii) freeze-drying the samples within the multiwell plates for 3 days; and (viii) punching dried polymeric networks into final shapes: a ring and a small disc; (ix) addition of a droplet of FN to the small disc followed by incubation at 37 °C for 1 h. This figure incorporates images adapted from Smart Servier Medical Art (https://smart.servier.com), provided under a Creative Commons Attribution 3.0 Unported License.

The presence of GG, HAp, and FN in dried polymeric networks was analyzed by FTIR (Figure 2A). GG exhibited the characteristic stretching vibrations at the region 3420 cm^–1^ (vO–H), 2920 cm^–1^ (vC–H), 1618 and 1412 cm^–1^ (vCOO−), and between 1060 and 1150 cm^–1^ (vC–O), as well as the bending vibrations at 1458, 1421, and 1373 cm^–1^ (vC–H), as previously documented.^34^ The presence of HAp was confirmed by the additional double peak at approximately 600 cm^–1^ (vPO43–, bending mode) and a peak at 960 cm^–1^ (vPO43–, stretching mode).^34^ The characteristic peaks of FN, specifically at 1659 cm^–1^ (vC=O, stretching), 1571 cm^–1^ (vN–H, deformation), and 1493 cm^–1^ (vC–N, stretching),^38^ were not clearly observed due to overlap with the GG peaks.

**Figure 2:**
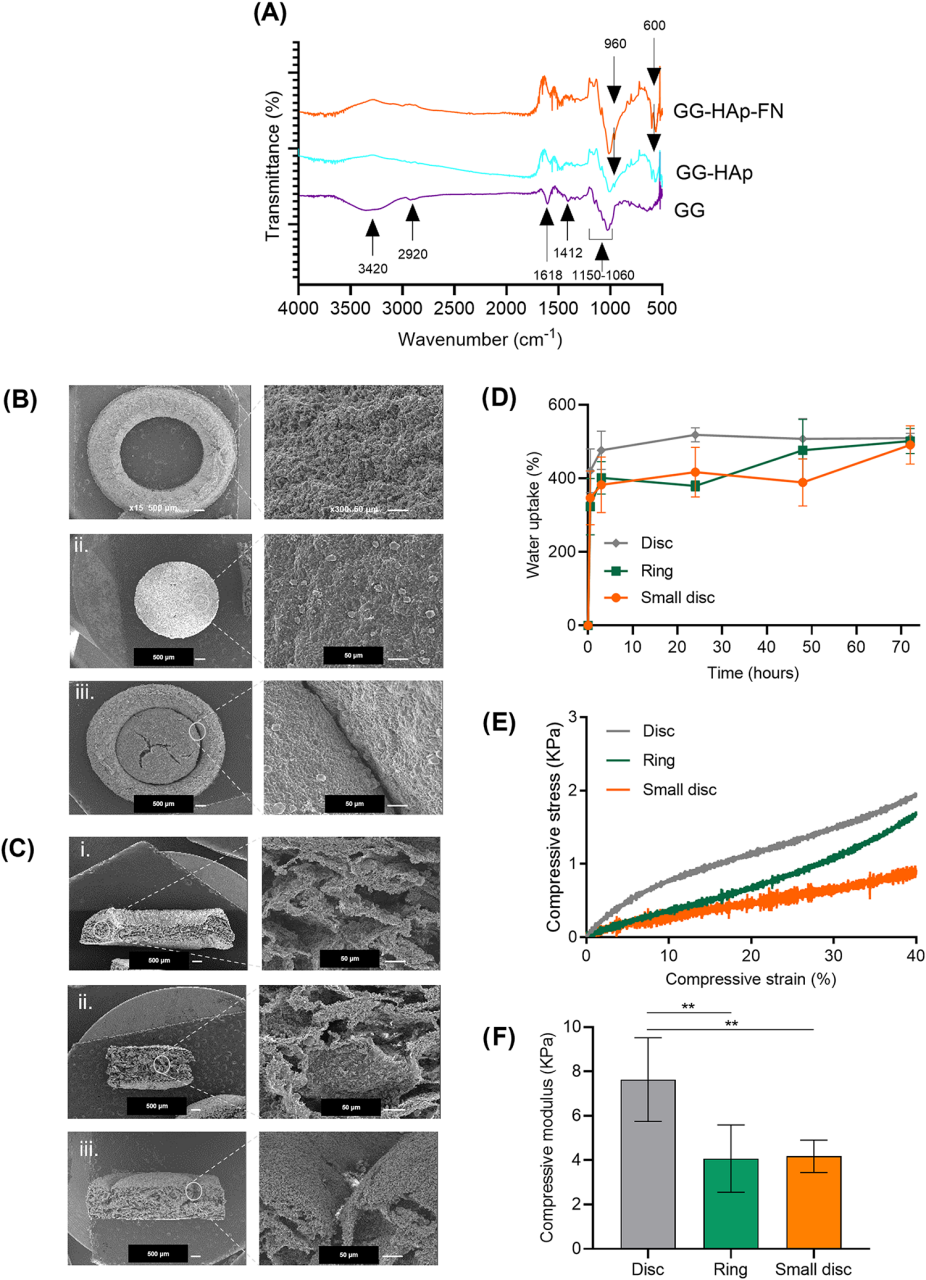
GG-HAp spongy-like hydrogel characterization. (A) FTIR spectra of FN-coated GG-HAp, GG-HAp, and GG spongy-like hydrogel small discs. (B) Top and (C) cross-sectional views of GG-HAp spongy-like hydrogels, prepared in various geometries (ring, small disc, 3D model, and control disc), analyzed by SEM. (D) Water uptake profile of GG-HAp spongy-like hydrogels, prepared in various geometries (ring, small disc, and control disc) over 3 days. (E) Stress–strain curves and (F) compressive modulus of GG-HAp spongy-like hydrogels measured in their initial disc form (control) and after being punched into the two geometries used in this study: rings and small discs. Measurements were taken following a 24 h incubation period in a PBS solution at 37 °C. All measurements were analyzed using one-way ANOVA and Tukey’s multiple comparisons *t* test. Significant difference was set at **p < 0.01, and data was presented by mean ± SD.

Dried polymeric networks were processed to have two parts that mimic the cortical-sponge bone architecture. In a sterile environment, both parts were obtained by centrally punching the dried polymeric networks with a 4 mm punch. This process yielded an 8 mm ring (with an inner diameter of 4 mm) and a small disc with a 4 mm diameter, both having a thickness of 3 mm (Figure S1). The obtained constructs can be used either together or separately, as showcased in Figure S1.ii. The outer ring was designed to replicate the architecture of cortical bone, while the small disc mimics the inner structure of sponge bone. The microarchitecture of the two parts was then analyzed by SEM (Figures 2B and 3C). HAp particles were entrapped within the polymeric pore walls and randomly but uniformly dispersed throughout the structure of the polymer matrix. The water uptake of GG-HAp dried polymeric networks, prepared in various geometries (ring, small disc, and control disc), was monitored over 3 days of immersion in PBS (Figure 2D). All formulations exhibited an initial burst of water uptake within the first hours of immersion, followed by a stabilization phase, indicating that the maximum water content had been reached. After 3 days of immersion, the GG-HAp spongy-like hydrogels achieved a water content ranging between 491.40 ± 52.30 and 509.50 ± 13.21. Comparable water content levels were observed across the other geometries. Additionally, the mechanical properties of GG-HAp spongy-like hydrogels were evaluated. As shown in Figure 2E, the strain–stress curves of GG-HAp processed in various geometries were analyzed. No fractures were observed, even at strain levels up to 40% of the strain. Furthermore, analysis of the compressive modulus (Figure 2F and Table S4) indicated that the compressive modulus of the disc-shaped GG-HAp spongy-like hydrogels was significantly higher (**, *p* < 0.01) compared to the spongy-like hydrogels shaped as rings and small discs. The disc-shaped GG-HAp spongy-like hydrogels exhibited a compressive modulus of 7.63 ± 1.89 KPa. In contrast, the spongy-like hydrogels shaped as rings and small discs had compressive moduli of 4.07 ± 1.52 and 4.18 ± 0.73 KPa, respectively.

**Figure 3:**
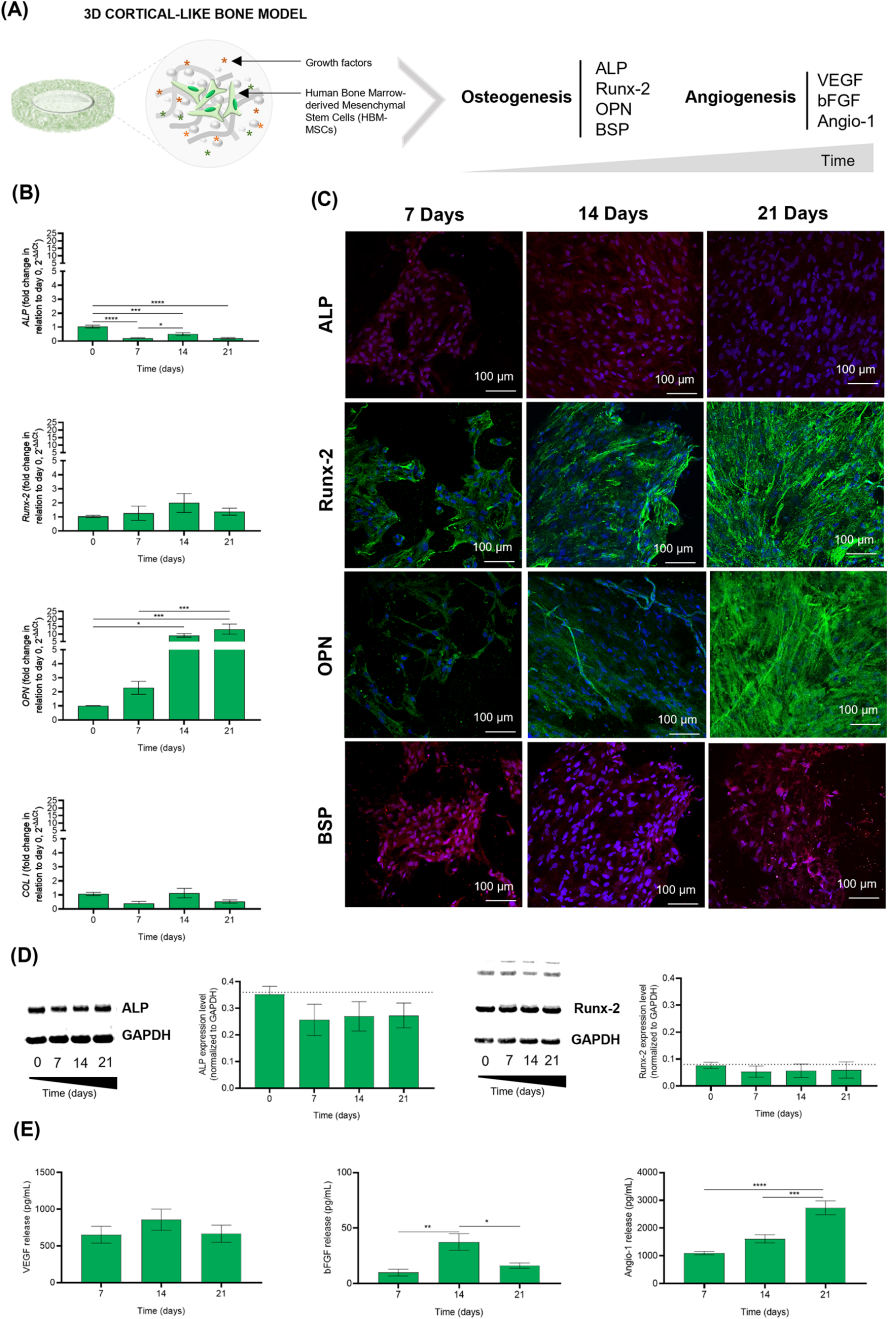
Establishment of a 3D cortical-like bone model. (A) Schematic representation of the 3D cortical-like bone model highlighting its essential elements such as GG, HAp, growth factors, and HBM-MSCs. Bone tissue maturation occurring in the 3D cortical-like bone model was analyzed by measuring the expression of osteogenic markers (*ALP*, *Runx-2*, *OPN*, and *BSP*). Its ability to release angiogenic growth factors (VEGF, bFGF, and Angio-1) was also evaluated. (B) Relative expression profile of ALP, Runx-2, *OPN*, and *COL I* genes in the 3D model along the culture. Graphics show the 2^–ΔΔCt^ value in relation to the expression of glyceraldehyde-3-phosphate dehydrogenase (GAPDH) and day 0 (30 min postseeding/entrapment). (C) Representative ALP, BSP, Runx-2, and OPN immunostained images, 7, 14, and 21 days postculture. Nuclei are counterstained with Hoechst (blue). (D) Relative expression of ALP and Runx-2 protein levels along the culture period, normalized by the housekeeping GAPDH protein, determined by WB. Representative blots are also displayed. (E) VEGF, bFGF, and Angio-1 secretion levels released by HBM-MSCs throughout the culture period, determined by Luminex assay. Data are presented as the mean ± standard error of the Mean (SEM). Statistical analysis was performed by a one-way ANOVA and Tukey’s multiple comparisons post-test. The significant differences were set at *p < 0.05, **p < 0.01, ***p < 0.001, and ****p < 0.0001.

Figure 3 illustrates the development of the 3D cortical-like bone model in which HBM-MSCs were cultured within the GG-HAp spongy-like hydrogel rings. After confirming cell viability within GG-HAp spongy-like hydrogel rings (Figures S2 and S3), the phenotype of HBM-MSCs within the GG-HAp spongy-like hydrogel rings was thoroughly analyzed (Figure 3A). The development of mature bone tissue was evaluated by measuring the expression of early (ALP, Runx-2, COL I, and BSP) and late (OPN) osteogenic markers as well as its capacity to release angiogenic growth factors (VEGF, bFGF, and Angio-1), important for vasculogenesis. The expression patterns of genes related to osteogenesis, namely, *ALP*, *Runx*-2, *OPN*, and *COL* I, are presented in Figure 3B, whereas the protein patterns are illustrated in Figure 3C and quantitatively analyzed in Figure 3D. The ALP gene exhibited statistically significant downregulation from day 0 to 7 (****, *p* < 0.0001), day 14 (***, *p* < 0.001), and day 21 (****, *p* < 0.0001) (Figure 3B and Table S5). This data was corroborated at the protein level (Figure 3D). Despite the ALP gene expression being downregulated (ALP fold change <1), it presented a significant increase at day 14 compared to 7 (*, *p* < 0.05). This increase in ALP expression was further confirmed by the higher ALP protein content observed on day 14, as evidenced by ICC analysis (Figure 3C) when compared to days 7 and 21. The Runx-2 gene expression was detected at all time points, displaying a peak in expression at day 14, although not statistically significant (Figure 3B). Protein expression confirmed the presence of Runx-2 at all time points (Figure 3C,3D). The OPN gene showed significant upregulation at day 14 (*, *p* < 0.05) and day 21 (***, *p* < 0.001) in comparison to day 0 (Figure 3B). Additionally, this significant upregulation was observed from day 7 to 21 (***, *p* < 0.001) (Figure 3B). This increase was also visualized by ICC (Figure 3C). Regarding COL I, it was detected only in terms of gene expression (Figure 3B,3C).

The secretion of vWF-A2, PDGF-BB, VEGF, bFGF, Angio-1, OPN, BMP-2, and BMP-4 by HBM-MSCs was quantified, as shown in Figure 3E. The proteins associated with angiogenesis (vWF-A2 and PDGF-BB) and osteogenesis (OPN, BMP-2, and BMP-4) were not detected in the cell culture supernatant throughout the entire culture period, likely due to their concentrations being below the detection threshold of the Luminex assay. However, the remaining angiogenic proteins studied (VEGF, bFGF, and Angio-1) were detected in cell culture supernatants along the time. Both VEGF and bFGF exhibited a similar release pattern, reaching a peak in secretion on day 14, followed by a decrease on day 21. While there were no significant differences observed in VEGF release, bFGF displayed a significantly higher release from day 7 to 14 (**, *p* < 0.001), followed by a significant decrease in release from day 14 to 21 (*, *p* < 0.05). In the case of Angio-1, it demonstrated a sustained increase in release throughout the culture period, with statistically significant differences observed from day 7 to 21 (****, *p* < 0.0001) and from day 14 to 21 (***, *p* < 0.001).

Figure 4 illustrates the development of the 3D sponge-like bone model, established through the coculture of HDMECs and HBM-MSCs within the GG-HAp spongy-like hydrogel small discs. First, we evaluated the optimal FN concentration for endothelial cell adhesion and determined it to be 10 μg/mL (Figure S3). We then tested the ability of ECs to form vascular-like structures on their own, but no such structures were observed (data not shown). Subsequently, experiments using different HDMECs/HBM-MSCs ratios (80:20 and 60:40) were conducted to assess their influence on vessel formation. The 80:20 ratio was found to be optimal, resulting in the formation of well-established and stable vascular networks (Figure S3). Then, we proceeded to assess the interaction between HDMECs and HBM-MSCs co-cultured within small discs of GG-HAp spongy-like hydrogel (Figure 4A). The development of the vascularized bone tissue was evaluated in terms of phenotype (CD31, VE-cadherin, and vWf), matrix composition (COL I and FN), and release of associated angiogenic factors (VEGF, bFGF, and Angio-1). In Figure 4B, positive CD31 immunostaining is presented as a prominent marker for ECs. On day 7, cellular organization begins to form toward a tubular-like structure, achieving a more mature state by day 14. This distinctive phenotype is reaffirmed at day 14 through the detection of additional endothelial cell-specific proteins, including VE-cadherin and vWf, via immunostaining. Additionally, the presence of essential ECM components, including an FN and COL I-enriched matrix, was validated using ICC, as depicted in Figure 4B, across the entire culture period.

**Figure 4:**
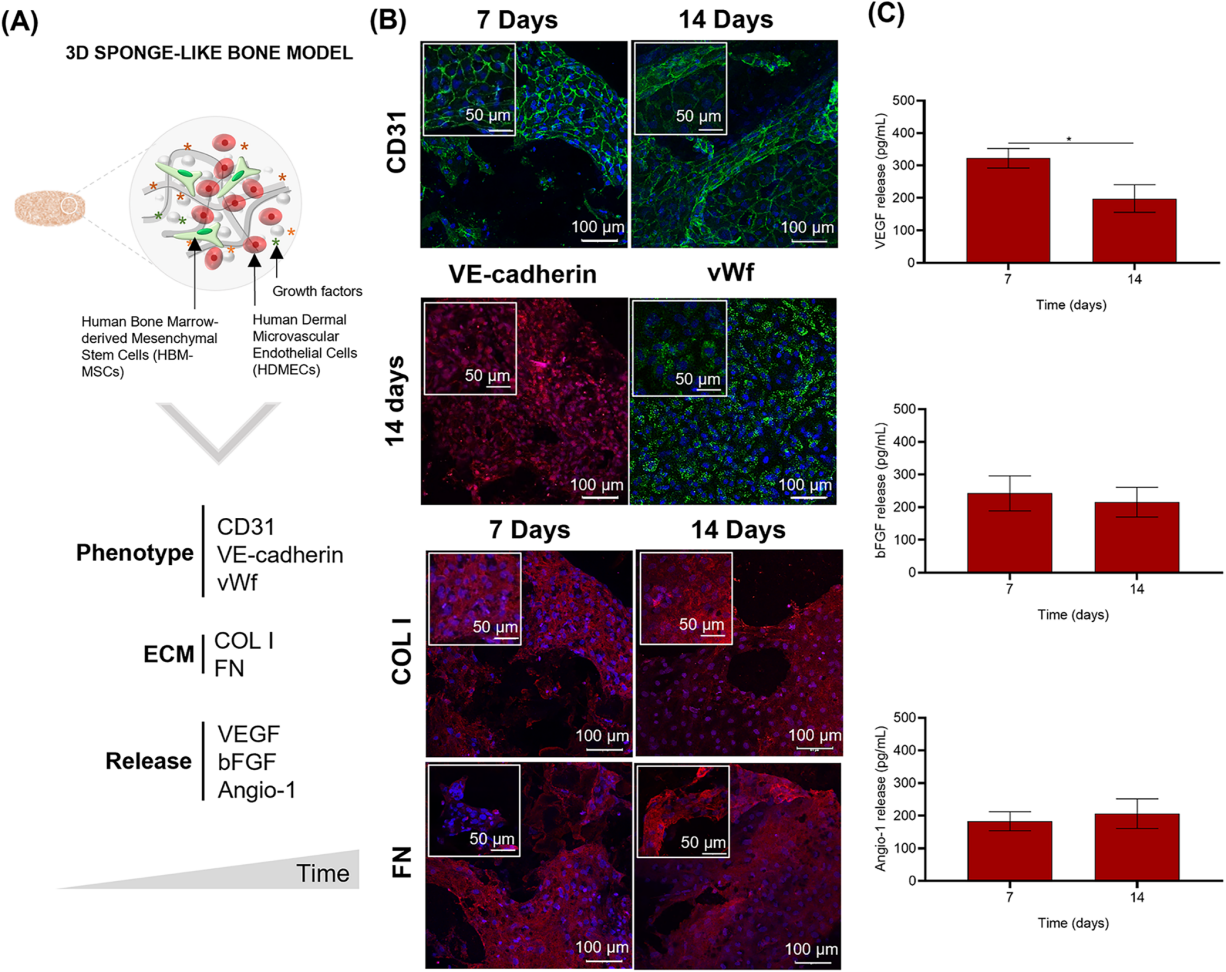
Establishment of a 3D sponge-like bone model. (A) Schematic representation of the 3D sponge-like bone model, integrating essential components like GG, HAp, growth factors, HBM-MSCs, and HDMECs. Vasculogenesis in the 3D vascularized bone was evaluated over a 14-day period by analyzing the expression of the typical endothelial phenotype markers (CD31, VE-cadherin, vWf), ECM proteins (COL I and FN), and the release of angiogenic growth factors (VEGF, bFGF, and Angio-1). (B) Representative CD31, VE-cadherin, vWf, COL I, and FN immunostained images up to 14 days postculture. Nuclei are counterstained with Hoechst (blue). (C) VEGF, bFGF, and Angio-1 levels secreted by cells laden in the 3D sponge-like bone model over 14 days, evaluated using a Luminex assay. Data were carried out in triplicate and presented as mean ± SEM. Statistical analysis was performed using an unpaired two-tailed Student’s *t* test, and the significant differences were set at *p < 0.05.

In Figure 4C, the secretion of vWF-A2, PDGF-BB, VEGF, bFGF, Angio-1, OPN, BMP-2, and BMP-4 was analyzed. As previously observed in the 3D cortical-like bone model, the proteins related to angiogenesis (vWF-A2 and PDGF-BB) and osteogenesis (OPN, BMP-2, and BMP-4) were not identified in the cell culture supernatant of the 3D sponge-like bone model. Although VEGF, bFGF, and Angio-1 were detected in cell culture supernatants, bFGF and Angio-1 showed a sustained release over the 14 days, but VEGF presented a higher secretion at 7 days, followed by a slight decline up to 14 days (*, *p* < 0.05).

To create the 3D cortical-sponge-like bone model, the 3D cortical-like and sponge-like bone models were placed in direct contact for a period of 7 days, giving rise to a 3D cortical-sponge-like bone model (Figure 5A). In detail, to evaluate the development of mature bone tissue with a vascularized network, the interaction between the two models at day 0 (with clearly defined boundaries) and after a 7-day period of direct contact, particularly the merging state/osteointegration, cell migration, and angiogenesis markers, was analyzed (Figure 5A). In Figure 5B, in agreement with the maturation of bone tissue, it is demonstrated that after 7 days of culture, the osteo-like part shows the presence of the osteogenic (ALP, Runx-2, OPN, and BSP)-related proteins. Furthermore, *ALP*, *Runx*-2, *COL* I, and *OPN* genes were also detected, although at a significantly lower amount than the 3D cortical-like bone model (without contact with the vascular-like part) (Figure 5C and Table S6). In this context, a significant downregulation was observed in the osteo-like part concerning *ALP* (*, *p* < 0.05), *Runx-2* (**, *p* < 0.01), *COL I* (**, *p* < 0.01), and *OPN* (**, *p* < 0.01) compared to the 3D cortical-like bone model. The WB data, aligned with the gene expression profile, revealed a downregulation in the expression of ALP and Runx-2 proteins in the osteo-like part when compared to the 3D cortical-like bone model, although this difference was not statistically significant. Regarding the vascular-like part (Figure 5E), tubular-like structures were detected, with cells showing positive staining for CD31, VE-cadherin, vWf, and FN deposition.

**Figure 5:**
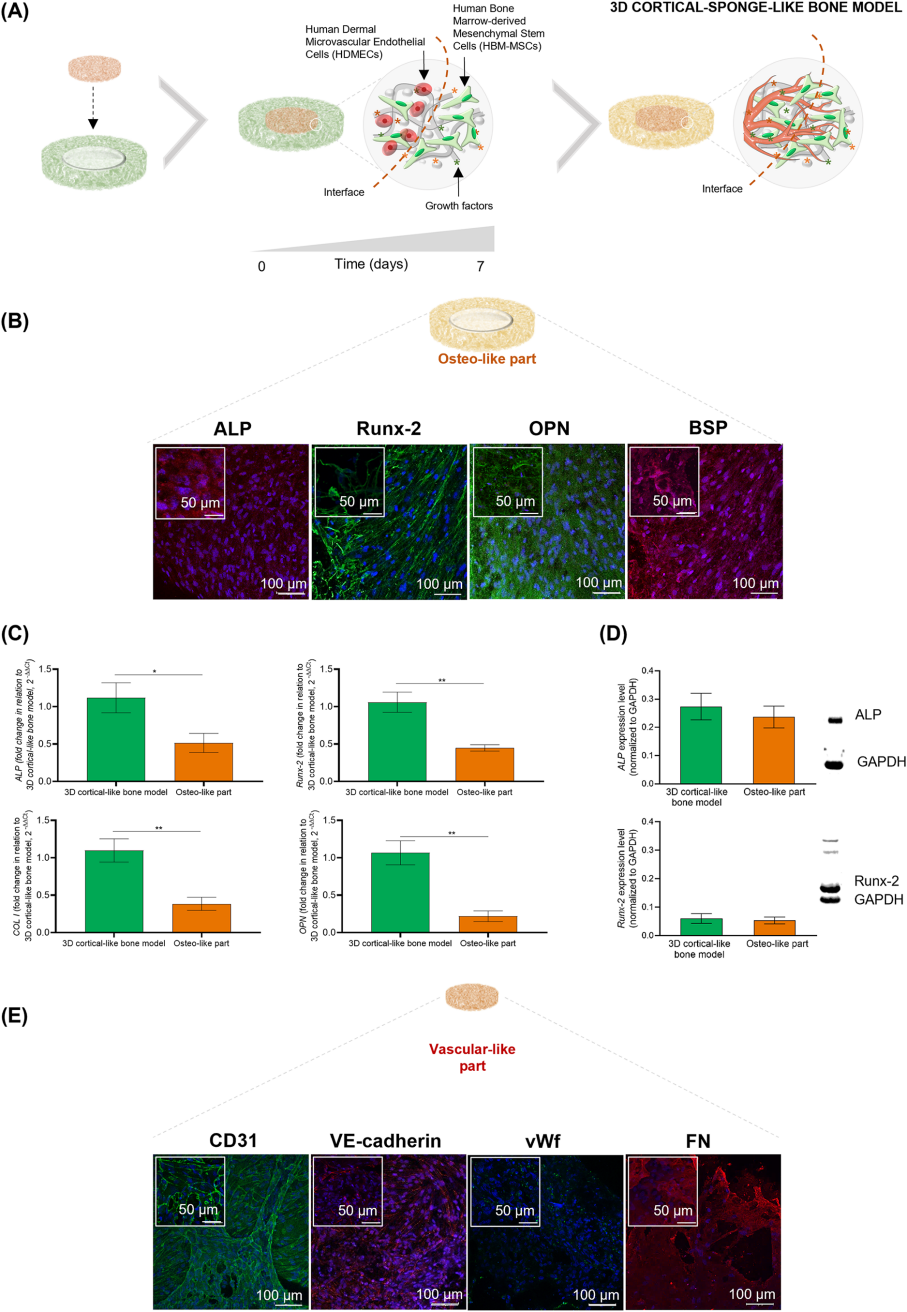
Establishment of a 3D cortical-sponge-like bone model. (A) Schematic illustration of the development of the 3D cortical-sponge-like bone model, where the osteo-like part was brought into direct contact with the vascular-like part (day 0), followed by a 7-day observation period. The assessment of osteointegration, cell migration, and angiogenesis was conducted on the mature and vascularized bone tissue resulting from a 7-day period of direct contact. (B) Representative ALP, Runx-2, OPN, and BSP immunostained images in the osteo-like part after 7 days of direct contact culture with the vascular-like part. Nuclei are counterstained in blue using Hoechst. (C) Relative expression profile of *ALP*, *Runx*-2, *OPN*, and *COL* I genes in the osteo-like part after 7 days of direct contact culture with the vascular-like part. Graphics show the 2^–ΔΔCt^ value relative to the *GAPDH* expression and normalized with respect to the 3D cortical-like bone model (cultured for the same time, 21 days). (D) Relative expression of ALP and Runx-2 protein levels in the osteo-like part after 7 days of direct culture with the vascular-like part, determined by WB. Graphics are normalized by the GAPDH housekeeping protein. Representative blots are also displayed. (E) Representative CD31, VE-cadherin, vWf, and FN immunostained images of the vascular-like part after 7 days of direct contact culture with the osteo-like part. Nuclei care counterstained in blue using Hoechst. All data are presented as the mean ± SEM and carried out in three independent experiments (*n* = 3). The statistical analysis was conducted using an unpaired two-tailed Student’s *t* test, and significance was established at *p < 0.05 and **p < 0.01.

Aiming to further analyze the bone-vascular coupling occurring between the bone-like and vascular-like parts, the intersection between the osteo and vascular-like parts was analyzed by ICC (Figure 6A). Vessels within the length range of small arteries (100–500 μm) occupied most of the biomaterial, with arterioles (10–100 μm) being predominant and sprouting from these larger vessels (Figure 6A­(i–ii)). Capillaries (≤10 μm) were also detected, albeit at lower levels. Particularly, vessels were found sprouting from the vascular-like part to the osteo-like part. A concomitant migration of mature bone cells was also evident from the osteo-like part to the vascular-like part, as affirmed by ALP-positive immunostaining (Figure 6A­(iii)). Figure 6B examines the release of vWF-A2, PDGF-BB, VEGF, bFGF, Angio-1, OPN, BMP-2, and BMP-4. Notably, proteins linked to angiogenesis (vWF-A2 and PDGF-BB) and osteogenesis (OPN, BMP-2, and BMP-4) were not found in the cell culture supernatant of the 3D sponge-like bone model. However, both cell types within the 3D cortical-sponge-like bone model secreted VEGF, bFGF, and Angiopoietin-1, which were further analyzed and compared to their respective controls—the 3D cortical-like bone model or the 3D sponge-like bone model—with similar culture periods (21 or 14 days, respectively). On the one hand, there was a significant increase in the secretion of VEGF (**, *p* < 0.01) and bFGF (***, *p* < 0.001) by the 3D cortical-sponge-like bone model in comparison to the 3D cortical-like bone model. Meanwhile, the Angio-1 levels secreted by the 3D cortical-sponge-like bone model were significantly lower (****, *p* < 0.0001) than the levels secreted by the 3D cortical-like bone model. On the other hand, the secretion of VEGF and Angio-1 was significantly higher (****, *p* < 0.0001) in the 3D cortical-sponge-like bone model in comparison to the 3D sponge-like bone model. Nevertheless, there were no differences in the release of bFGF between the vascular bone model and the 3D sponge-like bone model.

**Figure 6:**
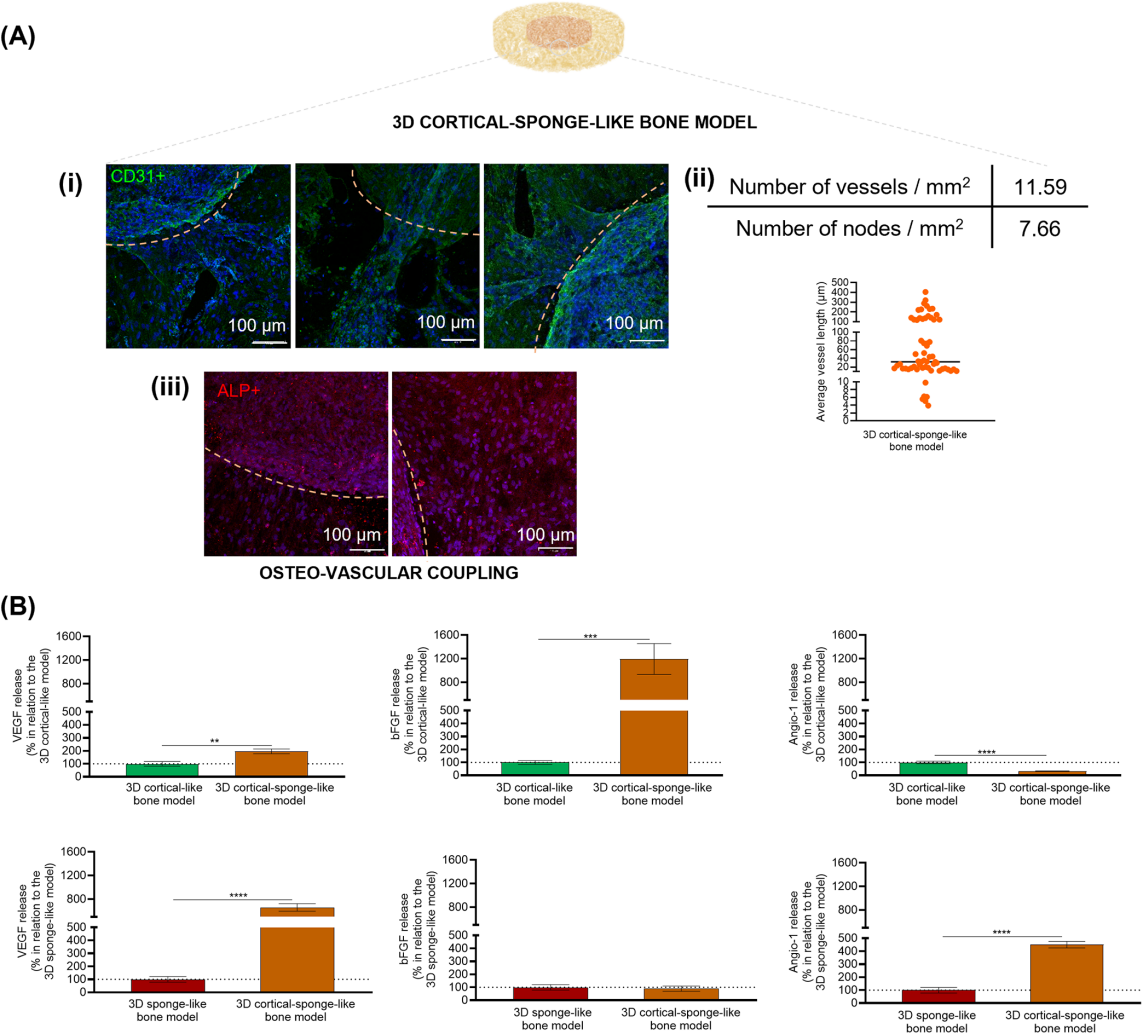
Osteo-vascular coupling within the 3D cortical-sponge-like bone model. (A) Representative CD31 and ALP immunostained images of the osteo-like and vascular-like intersections after 7 days of direct contact culture. An orange dashed line is designed to delineate the boundary between both parts. (B) VEGF, bFGF, and Angio-1 secreted from the 3D cortical-sponge-like bone model, evaluated using a Luminex assay. Data is presented as a percentage relative to their respective controls, specifically the 3D cortical-like bone model (cultured for the same time, 21 days) and the 3D sponge-like bone model (cultured for the same time, 14 days). The results are expressed as the mean ± SEM based on three independent experiments (*n* = 3). Absolute values in ppm are presented in Table S7. Statistical analysis was performed using an unpaired two-tailed Student’s *t* test, and significance was defined as **p < 0.01, ***p < 0.001, and ****p < 0.0001.

## 4 Discussion

Exploring innovative therapeutics using 3D bone *in vitro* models offers valuable insights into their effects and interactions within the bone microenvironment. Achieving an accurate representation of physiological responses observed in living organisms with these models requires a thorough grasp of bone anatomy and biology. Herein, we developed a 3D bone model designed to closely mimic the native biology of both cortical and sponge-like bone tissues. We aimed to go further with the replication of bone cortical and sponge structure and added the osteogenic and vascularized microenvironment by de novo recreating osteogenesis and vasculogenesis.

One of the main considerations while developing a reliable bone model consists of mimicking the organic and inorganic composition of the bone. Our previous studies^34–36,39^ demonstrated that GG-HAp spongy-like hydrogels not only mimic bone composition but also exhibit suitable microstructural properties. These include rugged surfaces, controlled water uptake, thicker pore walls, and pore sizes and porosities in accordance with literature standards, facilitating rapid nutrient and metabolite exchange. They are well-suited for engineering bone tissue models for testing potential therapies, advancing our understanding of bone biology, and showing the potential for addressing early stages of bone defects or fractures.

GG-HAp spongy-like hydrogels presented the characteristic mechanical properties of spongy-like hydrogels, with the ability to withstand applied loads, resist fracture, maintain flexibility, and recover rapidly under compressive stress. Their stiffness ranged between 4 and 7 KPa, which proved effective in supporting cell viability, adhesion, differentiation, and migration between both spongy and cortical-like parts. While this stiffness supported cellular functions, it does not align with the typical mechanical range of native bone tissue, which is in the gigapascal (GPa) range.^14,15^ Nonetheless, we anticipate that the cell-laden constructs will progressively develop a stiffer extracellular matrix that more closely resembles native *in vivo* conditions, driven by the matrix-producing activity of osteoblasts. Achieving stiffness closer to that of bone is still essential for promoting optimal cellular mechanosensing and mechanotransduction as well as mechanical performance under dynamic loading conditions. To overcome this current limitation, future work will focus on enhancing the polymer and HAp content to improve the mechanical properties of the spongy-like hydrogels, not only under static but also under dynamic environments, as previously explored by others.^7,40–42^

Bone anatomy, encompassing both cortical and trabecular components, also needs consideration when developing a bone surrogate. Aiming to recreate these different compartments, we processed the shape of the GG-HAp spongy-like hydrogel to have this anatomy. This was attained by punching a GG-HAp dried polymeric network disc into two different parts: a ring and a small disc. This processing methodology presents significant benefits for developing cortical-sponge bone models since these shapes can be used either independently to mimic the cortical (ring) or the sponge (disc) bone or together to mimic the cortical-sponge bone (the disc inside of the ring). Moreover, this approach is highly advantageous for creating cortical-sponge-like bone models, owing to its simplicity, cost-effectiveness, speed, and effectiveness. Unlike other studies that employ complex techniques to separately mimic the cortical and trabecular bone compartments—such as combining sponge replica with electrospinning, intricate 3D printing of complex structures, or using 3D extrusion-based printing with thermoplastic polymers and polymeric foam, as well as radial-gradient scaffolds with fractal designs—our approach shines for its straightforward preparation process.^22–24,28^

Bone mimicry further needs the integration of cellular components. Herein, we selected different cell types for growth within the different cortical and trabecular compartments. In the cortical-like part, we used HBM-MSCs and directed the cultured conditions to endorse osteogenesis, whereas, in the sponge/trabecular-like part, we used HDMECs supported by HBM-MSCs to endorse de novo vasculogenesis. The cortical bone mainly consists of osteocytes, but their isolation and accessibility is challenging due to difficulties in obtaining large quantities.^10^ In contrast, HBM-MSCs can be readily harvested and expanded *in vitro*. Additionally, they can be obtained directly from the patient, allowing for the development of personalized therapies using the patient’s own cells. Most importantly, by employing HBM-MSCs in the 3D cortical-like part, we can replicate intrinsic bone osteogenesis, since HBM-MSCs can undergo osteogenic differentiation to become osteoblasts.^43^ The trabecular bone distinguishes itself from cortical bone by the presence of a vascular network.^44^ Recognizing this, we hypothesized that by integrating HDMECs within the 3D sponge-like part, we would recreate the intricate vascular network crucial for delivering nutrients and oxygen to tissues. On one side, HDMECs offer another avenue for personalized medicine, as they can be harvested from a small skin biopsy, facilitating the development of vascularized constructs customized to each patient’s requirements.^45^ On the other side, HDMECs are more physiologically relevant for vasculogenesis compared to other commonly used endothelial cells such as HUVECs, particularly when modeling tissue-specific capillary networks (e.g., skin or bone).^45^ Acknowledging the limited adhesion of HDMECs to spongy-like hydrogels,^37^ we coated biomaterials with FN to enhance it, as previously demonstrated by us. Moreover, since establishing an effective and functional vascularized network requires the presence of supporting cell types, like pericyte cells, fibroblasts, and smooth muscle cells,^46^ we included HBM-MSCs as the first group of supporting cells. HBM-MSCs are not only known to contribute directly to tube-like structure formation but also indirectly by secreting VEGF.^43^ The accessibility of HDMECs, coupled with the versatility and regenerative potential of MSCs, exemplifies a personalized medicine approach wherein patient-specific cells are employed to craft customized 3D *in vitro* models. Future approaches may explore incorporating additional supportive cell types to further advance the model’s development.

With the experimental steps well-defined, we evaluated the feasibility of inducing cortical-like bone formation and maturation within the standard culture duration of osteogenic differentiation (21 days). Osteogenic differentiation of MSCs into osteoblasts is typically induced *in vitro* using a cocktail containing dexamethasone, β-glycerophosphate, and ascorbic acid and unfolds over approximately one month.^47,48^ This progression involves a tightly regulated sequence of transcription factor activation and ECM protein synthesis. Early in this process, around the first to second week, cells reduce proliferation and begin expressing early osteogenic markers such as ALP and Runx-2, along with producing COL I. As differentiation progresses, increased levels of OPN expression and the continued presence of COL I reflect ongoing matrix deposition and the progression of mineralization. ALP remains a key marker throughout, given its strong association with mineralized tissue and its essential role in bone matrix calcification.^49^ Consistent with this well-characterized progression, we observed an increase in all osteogenic-related genes (ALP, Runx-2, OPN, and COL I) by day 14 of culture, although not statistically significant across all genes. In more detail, ALP was upregulated shortly after seeding, indicating an early commitment to osteogenesis. ALP is a crucial phenotypic indicator released by osteoblasts, typically showing an initial rise during the early stages of osteoblast differentiation.^50^ This increase persists during osteoblast maturation, aligning with the observations in our study of 14 days.

Also, the slight increase of the early osteogenic marker—Runx-2—up to day 14 suggests that by this time, the majority of HBM-MSCs have differentiated into preosteoblasts and immature osteoblasts.^51^ While the decrease in expression by day 21 may lack statistical significance, it indicates a commitment to the fully matured stage. Additionally, the slight increase in COL I expression at 14 days and the significant increase in OPN expression, particularly at 14 and 21 days, reinforces the ECM maturation during the culture period.^52^ These results were consistent with other studies that showed the influence of HAp-containing scaffolds on regulating osteogenic genes, leading to a more pronounced upregulation of genes linked to osteogenic differentiation.^53–55^ Accordingly, a time-dependent increase in the expression of osteogenic genes (such as *ALP*, *Runx*-2, *OPN*, and OC), was observed at later time points, 14 days, compared to earlier time points (e.g., 7 days). This highlights the progressive enhancement of osteogenic differentiation over time. Given that, we inferred that by the 14th day of culture, we achieved a mature bone tissue, which we denoted as the 3D cortical-like bone model. These observations align with the expected osteogenic timeline and underscore the importance of capturing dynamic changes across specific time points.

In light of these findings, we further aimed to develop the 3D sponge-like bone model. One of the goals was to achieve a vascularized tissue that physiologically exists in the sponge bone. In line with this rationale, we assessed the phenotype, matrix composition, and angiogenic capacity in the sponge-like bone model up to 14 days of culture. After 7 days, cells started arranging into tubular-like structures, along with an increased deposition of COL I and FN within the ECM. This was concomitant with a significant release of the pro-angiogenic factor VEGF at 7 days, suggesting its potential contribution to the observed structural organization. Meanwhile, bFGF and Angio-1 exhibited sustained release throughout the culture period. In addition to VEGF, Angio-1 is also known to foster endothelial cell survival, promote migration, and facilitate the formation and stabilization of capillary-like structures,^56^ while bFGF is responsible for increased angiogenic sprouting capacity, playing a crucial role in the growth and differentiation of ECs and MSCs.^46^ After 14 days of culture, cells expressed VE-cadherin and vWf, indicative of the maturation and functional integrity of ECs, which are essential for the formation and stabilization of blood vessels. These markers were observed by other authors only in the later stages of culture, specifically at 21 days.^57^ Given that, we inferred that by the seventh day of culture, we achieved a prevascularized tissue, which we denoted as the 3D sponge-like bone model.

The following step included the direct-contact culture of both the cortical-like and sponge-like bone models to establish a 3D cortical-sponge-like model. We assembled the 3D cortical-like bone model cultured individually for 14 days for osteogenic differentiation with the 3D sponge-like model cultured individually for 7 days while reaching its prevascularized state. The sponge-like part perfectly fitted into the cortical-like part, suggesting no signs of degradation over the culture period. Moreover, after 7 days of culture, no signs of degradation or shape alteration were noticed, and both the small disc and ring remained structurally intact and well-integrated. In fact, degradation of spongy-like hydrogels was not expected, as previous studies of *in vitro* degradation over a 28-day period indicated minimal degradation, with weight loss less than 20%.^34^ This integration was further confirmed by the cell–cell interaction between the cells of the inner and outer parts of the 3D cortical-sponge-like bone model. A simultaneous migration of both cell types from one part to the other, revealing tubular-like structures and sprouting from the vascular-like part into the osteo-like part, was observed. It suggests that osteoblast-like cells differentiated from HBM-MSCs in the bone-like part have recruited ECs. In fact, vessels in the ranges of small arteries (100–500 μm) and arterioles (10–100 μm) were predominantly found in the model. Moreover, a high release of VEGF, bFGF, and Angio-1 was detected along the culture period in individual cortical-like bone models and with an even higher release in assembled cortical-sponge-like bone models. The latter emphasizes the synergistic influence of the direct contact co-culture model, highlighting an enhanced pro-angiogenic potential. These results align with those of Payal Ganguly and co-authors, who developed a 3D construct fostering an environment conducive to angiogenesis by co-culturing MSCs and ECs, thereby promoting VEGF production.^58^ Nonetheless, a significant downregulation of *ALP*, *Runx*-2, *COL* I, and *OPN* genes was detected in the cortical-like part of the assembled model compared to the individual cortical-like bone model, suggesting that angiogenesis took precedence over osteogenesis. In fact, a complex and dynamic interplay between vasculogenesis and osteogenesis occurs during the early stages of tissue development. This observation is consistent with the well-documented concept that vasculogenesis often precedes and supports osteogenesis by establishing a vascular network that delivers essential oxygen, nutrients, and signaling molecules. However, ECs can also regulate osteogenic differentiation through shared signaling pathways, such as BMPs, potentially influencing HBM-MSCs’ fate by improving osteogenesis or activating feedback inhibition mechanisms.^59^ Although most literature indicates a positive effect in osteogenesis,^59^ it has been recently demonstrated that mice with endothelial cell-conditional SMAD1/5 depletion show hypervascularity and altered trabecular and cortical bone formation,^60^ supporting our results. Moreover, HUVEC-MSC co-cultures have been shown to reduce mineralization under certain conditions.^46^ This suggests that the early emphasis on angiogenic signaling in our model may have transiently suppressed osteogenesis. ALP immunostaining, indicative of osteogenic differentiation and their conducive microenvironment for bone mineralization, growth, and formation, was still detected in the osteo-like part. Interestingly, the vascular-like part also showed positive ALP immunostaining, suggesting that MSCs in the vascular part were influenced by the osteogenic cortical-like part and/or the osteogenic culture conditions throughout the 7 days of culture. This interplay between the osteogenic and vascular components further underscores the plasticity and polyvalent capabilities of MSCs, as also demonstrated in other studies focused on generating functional microvascular networks for tissue-engineered bone grafts.^61^ This observation highlights the potential for optimizing the balance between vasculogenesis and osteogenesis in future studies to enhance the functional outcomes of the 3D bone model.

## 5 Conclusions

In conclusion, our study effectively created a 3D cortical-sponge-like bone model, emphasizing the essential contributions of osteogenesis and vascularization to the development of reliable 3D models for studying bone diseases. Through the engineering of a versatile 3D *in vitro* model using GG-HAp spongy-like hydrogels, we replicated the hierarchical biological microarchitecture of bone, demonstrating successful tissue maturation and vascularization in both cortical-like and sponge-like components, respectively. Employing HBM-MSCs in the 3D cortical-like bone model, we replicate the natural process of bone osteogenesis, wherein these cells differentiate into osteoblast-like cells, as demonstrated by the expression of osteogenic markers. Additionally, our study observed cellular organization toward tubular-like structures in the 3D sponge-like model, indicating signs of vasculogenesis supported by crucial angiogenic growth factors, endothelial cell-specific proteins, and essential extracellular matrix components. Upon achieving maturity in osteogenesis and the formation of capillary-like structures, the integration of both constructs generated the 3D vascular-bone-like model, fostering an environment conducive to the coupling of osteogenesis and vasculogenesis, closely resembling native bone characteristics *in vitro*. This innovative approach enables the exploration of osteo-vasculogenesis coupling by integrating vascular attributes into the cortical-like bone model, which is crucial for advancing our comprehension of bone physiology and diseases and for fabricating tissue-engineered constructs that faithfully replicate bone tissue.

## Supplementary Material



## Notes

Additional experimental details, including primer’s sequence list (Table S1), primary and secondary antibodies list (Tables S2 and S3), summary of compressive modulus (Table S4), gene expression fold changes in the 3D cortical-like model along the time (Table S5), gene expression fold changes in the 3D cortical-like bone model and osteo-like part of the 3D cortical-sponge-like bone model, after 7 days of coculture (Table S6), and angiogenic factors (pg/mL) released from the 3D cortical-like bone model, the 3D sponge-like bone model, and the 3D cortical-sponge-like bone model, after 7 days of coculture (Table S7), representative images of dried polymeric networks processing into cortical-like and sponge-like bone parts (Figure S1), representative images of live/and dead cells within GG-HAp spongy-like hydrogels with/without fibronectin coating at different concentrations (Figure S2), and representative images of vascular structure formation within GG-HAp spongy-like hydrogels after 10 days of culture (Figure S3) (PDF)
