# Supplementary material for: Bioinspired 3D Bone Model: Mimicking the Cortical–Spongy Bone Architecture and Biology for Enhanced Physiological Representation of Bone
Source: ACS Biomater Sci Eng. 2025 Jul 9;11(8):4915–30. doi: 10.1021/acsbiomaterials.5c00326 (PMC12820933; doi:10.1021/acsbiomaterials.5c00326)
Supplement: Supplementary file 1 [file ab5c00326_si_001.pdf]

A Bio-Inspired 3D Bone Model: Mimicking Cortical-Sponge Bone Architecture and Biology for Enhanced Physiological Representation of Bone

Ana R. Bastos [1,2], Lucília P. da Silva [1,2]*, Rui L. Reis [1,2], Vitor M. Correlo [1,2]*

[1] 3B's Research Group, I3Bs - Research Institute on Biomaterials, Biodegradables and Biomimetics, University of Minho, Headquarters of the European Institute of Excellence on Tissue Engineering and Regenerative Medicine, AvePark, Parque de Ciência e Tecnologia, Zona Industrial da Gandra, 4805-017 Barco, Guimarães, Portugal

[2] ICVS/3B's - PT Government Associated Laboratory, Portugal

*Corresponding authors: Lucília P. da Silva (E-mail: lucilia.silva@i3bs.uminho.pt); Vitor M. Correlo (E-mail: vitorcorrelo@i3bs.uminho.pt)

Supporting Information

1. Materials and Methods

Table S1. Primer's sequence list of bone-related genes.

Genes	Sequences	
	Forward (5' – 3')	Reverse (3'- 5')
ALP	CTCCTCGGAAGACACTCTG	AGACTGCGCCTGGTAGTTG
Runx-2	TTCCAGACCAGCAGCACTC	CAGCGTCAACACCATCATTC
OPN	CCCACAGACCCTTCCAAGTA	GGGGACAACTGGAGTGAAAA
COL I	AAGAACCCAAGGACAAGAG	GTAGGTGATGTTCTGGGAGG
GAPDH	ACAGTCAGCCGCATCTTCTT	GACAAGCTTCCCGTTCTCAG

Table S2. Primary and secondary antibodies used in the ICC analysis.

Antibodies	Dilution	Reference
Alkaline Phosphatase (Tissue Non-Specific) antibody	1 : 200	GTX100817
Anti-RUNX2 Antibody, clone AS110	1 : 50	05-1478
Osteopontin antibody [7C5H12]	1 : 100	GTX60672
Collagen I Antibody	1 : 100	NB600-408
Anti-Bone Sialoprotein II Antibody	1 : 200	AB1854
Goat Anti-Mouse IgG H&L (Alexa Fluor® 488)	1 : 500	AB150113
Donkey Anti-Rabbit IgG H&L (Alexa Fluor® 594)	1 : 500	AB150076

Table S3. Primary and secondary antibodies used in WB analysis.

Antibodies	Dilution	Molecular Weight (kDa)	Reference
**Alkaline Phosphatase (Tissue Non-Specific) antibody**	1: 2000	57	GTX100817
**Anti-RUNX2 Antibody, clone AS110**	1:1000	54-57	05-1478
**Rabbit monoclonal [EPR16891] to GAPDH**	1:10 000	36	ab181602
**IRDye® 680RD Goat-anti-Mouse Antibody**	1:10 000	-	926-68070
**IRDye® 800CW Goat-anti-Rabbit Antibody**	1:10 000	-	926-32211

Table S4. Compressive Modulus of GG-HAp spongy-like hydrogels. Results are presented as kPa. Results are expressed as mean ± S.D. and based on six independent experiments (n=6).

	Disc	Ring	Small Disc
**Compressive Modulus (kPa)**	7.632 ± 1.889	4.073 ± 1.516	4.175 ± 0.7298

Table S5. Gene expression in the 3D cortical-like model along the time. Results are presented as fold changes in relation to day 0. Results are expressed as mean ± S.D. and based on three independent experiments (n=3).

	Day 0	Day 7	Day 14	Day 21
**ALP**	1.042 ± 0.1361	0.193 ± 0.1361	0.499 ± 0.3085	0.198 ± 0.1504
**Runx-2**	1.029 ± 0.2501	1.260 ± 1.518	1.996 ± 2.039	1.369 ± 0.7470
**OPN**	1.003 ± 0.0737	2.291 ± 1.384	9.101 ± 3.161	13.260 ± 9.609
**COL I**	1.060 ± 0.3499	0.394 ± 0.4537	1.130 ± 1.010	0.527 ± 0.3581

Table S6. Gene expression in the osteo-like part of the 3D cortical-sponge-like bone model, after 7 days of co-culture. Results are presented as fold changes in relation to the 3D cortical-like bone model independently cultured for the same period (21 days). Results are expressed as mean ± S.D. and based on three independent experiments (n=3).

	3D cortical-like bone model	Osteo-like part
**ALP**	1.120 ± 0.6022	0.5156 ± 0.3825
**Runx-2**	1.060 ± 0.4022	0.4489 ± 0.1278
**OPN**	1.068 ± 0.4539	0.2200 ± 0.2170
**COL I**	1.099 ± 0.4659	0.3856 ± 0.2619

Table S7. Angiogenic factors released from the 3D cortical-sponge-like bone model after 7 days of co-culture, as well as from the 3D cortical-like bone model and the 3D sponge-

like bone model independently cultured for the same period (21 days and 14 days, respectively). Results are presented as pg/mL. Results are expressed as mean ± S.D. and based on three independent experiments (n=3).

	3D cortical-like bone model	3D sponge-like bone model	3D cortical-sponge-like bone model
**VEGF**	617.6 ± 341.1	187.6 ± 125.6	1392 ± 287.7
**FGF-b**	16.24 ± 7.502	203.8 ± 140.7	183.1 ± 132.0
**Angio-1**	2608 ± 699.7	222.9 ± 128.5	930.5 ± 165.8

Results

Dried polymeric networks were processed to have two parts that mimic the cortical-sponge bone architecture. In a sterile environment, both parts were obtained by centrally punching the dried polymeric networks with a 4 mm punch. This process yielded an 8 mm ring (with an inner diameter of 4 mm) and a small disc with a 4 mm diameter, both having a thickness of 3 mm (Figure S1). The obtained constructs can be used either together or separately, as showcased in Figure S1.ii.

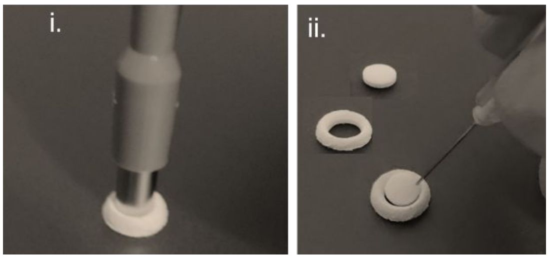

Figure S1. Representative images of dried polymeric networks processing into cortical-like and sponge-like bone parts.

Live and dead cells within GG-HAp spongy-like hydrogels, with or without fibronectin coating at different concentrations, were evaluated (Figure S2). Cells were mostly live in all conditions, except in biomaterials coated with 100 µg/mL. The optimal fibronectin concentration for endothelial cell adhesion and determined it to be 10 µg/mL (Figure S2).

Conditions	Cell type	Coating	Total Cell Density	Medium
**A**	HDMECs	Fibronectin (10 µg/mL)	800 000 cells/scaffold	Basal medium (EGM-2MV)
**B**	HDMECs	Fibronectin (100 µg/mL)	800 000 cells/scaffold	Basal medium (EGM-2MV)
**C**	HBM-MSCs	–	260 000 cells/tube	DMEM

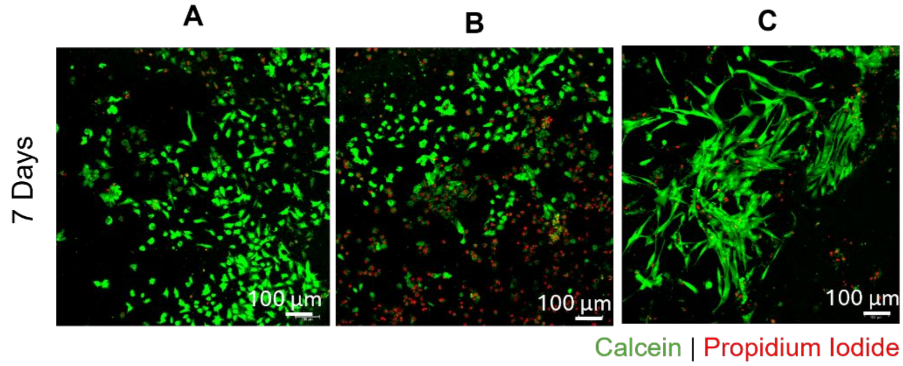

Figure S2 – Live (calcein AM staining) and dead (propidium iodide staining) cells within GG-HAp spongy-like hydrogels with or without fibronectin coating at different concentrations.

Vascular structure formation (CD31 immunostaining) within GG-HAp spongy-like hydrogels were evaluated (Figure S3). Since HDMECs cultured alone did not form any vascular structures (data not shown), the impact of co-culture on vessel formation, at HDMEC:HBM-MSC ratios of 80:20 and 60:40, were tested. The 80:20 ratio proved to be optimal, leading to the formation of well-defined and stable vascular networks (Figure S3).

Conditions	HDMECs : HBM-MSCs (%)	Total Cell Density	Medium
**A**	80 / 20	1 000 000 cells	Basal medium (EGM-2MV)
**B**	60 / 40	1 000 000 cells	Basal medium (EGM-2MV)

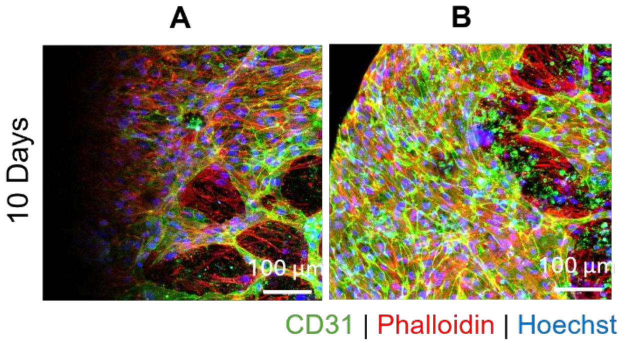

Figure S3 – Vascular structure formation (CD31 immunostaining) within GG-HAp spongy-like hydrogels after 10 days of culture.
